# Initial experience of ureteric visualization using methylene blue during laparoscopy for gynecological surgery

**DOI:** 10.3389/fsurg.2024.1387038

**Published:** 2024-07-18

**Authors:** Ruyu Shao, Faquan Shen, Hooman Soleymani majd, Xiaoqing Qin, Desheng Yao, Ying Long, He Wang, Yousheng Wei, Xin Chang

**Affiliations:** ^1^ Department of Gynecologic Oncology, Guangxi Medical University Cancer Hospital, Nanning, Guangxi, China; ^2^ Nuffield Department of Women’s & Reproductive Health, University of Oxford, Oxford, United Kingdom

**Keywords:** gynecological surgery, methylene blue, ureter, iatrogenic ureteral injury, laparoscopic surgery

## Abstract

**Objectives:**

Iatrogenic ureteral injury is a severe surgical complication, with a highest incidence of 1.5% in gynecological surgeries. The purpose of this report is to document our initial experience with using methylene blue (MB) to label the ureter in gynecological laparoscopic surgeries and to explore its effectiveness and safety. This is also a novel description of simultaneously visualizing ureteral MB fluorescence and sentinel lymph nodes (SLN's) Indocyanine Green (ICG) fluorescence using the same camera.

**Methods:**

This study included patients undergoing gynecological laparoscopic surgeries, with the same surgeon performing all cases. During the early stages of each surgery, rapid intravenous infusion of MB was administered. For cases requiring SLN imaging, we also injected ICG solution into the cervix. Assessment of the included cases was conducted both intraoperatively and postoperatively. The group that had MB fluorescence (Group A) was compared to a control group that did not have it (Group B).

**Results:**

A total of 25 patients (Group A) received MB during surgery, demonstrating 45 ureters clearly, with an imaging success rate of 90%. Continuous and clearer fluorescence imaging was achieved in cases with ureteral hydronephrosis. In most patients, ureteral fluorescence was visible 15–20 min after intravenous infusion of MB, and 64% still exhibited fluorescence at the end of the surgery. In patients who had both ICG and MB, dual fluorescence imaging was achieved clearly. Among the included cases, there were no iatrogenic ureteral injuries (0%), which we observed to be lower than in patients who did not receive MB (1.3%). The rate of adverse events was similar in both groups.

**Conclusion:**

Using MB fluorescence is an effective and safe method of visualizing the ureters during gynecological surgeries, and can diminish iatrogenic ureteral injury without increased associated adverse events. It therefore may offer promising prospects for clinical application.

## 1 Introduction

The ureters are structures that surgeons need to pay close attention to during pelvic and abdominal surgeries. Iatrogenic ureteral injury is uncommon, but it can lead to serious complications, including loss of renal function (1). The ureters have a close anatomical relationship with the female reproductive system, and 41.9% of iatrogenic ureteral injuries occur during gynecological surgeries (2). The distal segment of the ureter is the most vulnerable, with over 80% of surgical injuries occurring in this anatomical region (3). In gynecological surgeries, ureteral injuries primarily occur during procedures such as hysterectomy and pelvic tumor surgeries (4) This is especially risky in complex and challenging pelvic surgeries involving conditions like deep Infiltrating endometriosis (DIE)-induced adhesions, large tumors, cervical fixation, and tumor infiltration. If ureteral injury is not promptly identified and repaired during surgery, the consequences are patient morbidity which could include ureteral obstruction, fistulas and irreversible kidney damage. Therefore, the prevention and early intraoperative detection of ureteral injury are crucial for patients’ outcomes. Ureteral stents have been used for intraoperative identification to help reduce iatrogenic ureteral injury. However, this practice increases surgical time and costs, and may lead to urinary complications such as infection, hematuria, and perforation (5, 6). Some studies have instead explored the use of radiopaque agents for ureteral identification. However, this technique exposes both patients and healthcare personnel to ionizing radiation and lacks real-time visual information, and hence is unable to provide immediate guidance during surgery (7). Another report has proposed the use of Indocyanine Green (ICG) to mark the ureteral branch of the uterine artery, enabling preservation of the ureteral branches during radical hysterectomy (8).

Methylene blue (MB) is both a dye and a fluorescent substance. During surgery, due to its blue staining characteristics, it is used to assess anastomotic leaks. With the development of near-infrared imaging systems, the near-infrared fluorescent properties of MB have been utilized for applications such as breast lymph node localization (9), vascular imaging, and bile duct imaging (10), all achieving favorable clinical results. After intravenous administration of MB, the majority undergoes renal metabolism and is ultimately excreted in the urine.

In this study, patients deemed to be prone to ureteral injury during surgery received intravenous MB and we utilized near-infrared fluorescence imaging technology to visualize ureters in gynecological surgeries. We also introduced the simultaneous use of ICG and MB for fluorescence imaging in cervical cancer and endometrial cancer surgeries, where ICG was used for visualizing sentinel lymph nodes (SLN) and MB was for ureteral imaging. This was our first instance of displaying two types of fluorescence using the same camera. And we conducted a preliminary assessment of the feasibility and safety of real-time ureteral localization using MB in gynecological surgeries. During the same period, some patients declined the use of MB, and the same surgeon proceeded to perform their surgeries without it. We preliminarily compared the adverse events between these patients and those who had received MB.

## 2 Material and methods

This study was approved by the Institutional Review Board of the Guangxi Medical University Cancer Hospital. This study required patients to be over 18 years old and qualified for laparoscopic surgery for the removal of the uterus or pelvic lesions. Pregnant or lactating individuals, those with severe renal insufficiency, complete ureteral obstruction, G-6-PD deficiency, or known allergy to MB were excluded from this study. All patients provided signed informed consent before the surgery, and all these surgeries were performed by the same surgeon.

### 2.1 Fluorescence imaging equipment

Dual-mode laparoscopic fluorescence image system (Guangdong OptoMedic, Guangdong, China) was used in this study. This device is capable of simultaneously exciting two near-infrared lights, allowing MB to display purple-blue fluorescence and ICG to display green fluorescence. The light source module uses 660 nm and 805 nm light as the excitation light for MB and ICG. During fluorescence imaging, the light source module simultaneously outputs white light illumination and near-infrared excitation light. The camera module captures both the reflected white light information from the observed tissue and the fluorescence information excited by the contrast agent, generating white light and fluorescence images. These images are processed by the image processing unit, and the final output includes white light, fluorescence, and composite images, providing fluorescence navigation information for the surgery.

The fluorescence imaging system offers three fluorescence modes: ICG fluorescence mode, MB fluorescence mode, and dual fluorescence mode (ICG and MB fluorescence). It can simultaneously display white light images and fluorescence images for each mode on the same screen.

### 2.2 Methods and assessment

All surgeries were performed laparoscopically, and a dose of 1.5 mg/kg MB (Jiangsu Jichuan, Jiangsu, China) was added in 250 ml 5% glucose solution. At the beginning of surgeries, MB solution was rapidly infused intravenously. For cases requiring SLN imaging, such as cervical cancer and endometrial cancer, we additionally injected ICG solution (2.5 mg/ml) (Dandong Yichuang, Dandong, China) at the cervix's 3 and 9 o'clock positions after the instruments entered the abdominal cavity, with injection depths of 0.2 cm, 0.5 cm, and 1.0 cm, each side receiving 1 ml. During the surgeries, we observed both sides of the pelvic ureters in white light and fluorescence modes. The recommended observation position was the segment where the ureter crossed the iliac artery. Based on the patient's urine output and ureteral imaging, 20 mg of furosemide was administered intravenously if necessary to increase urine output. Concurrently, we monitored vital signs of patients, and compared renal function before and after surgeries. Adverse events after surgery were also collected.

## 3 Results

We included a total of 25 patients who underwent intraoperative ureteral MB fluorescence imaging at the Department of Gynecologic Oncology, Guangxi Medical University Cancer Hospital, from February 2022 to December 2022. Among them, there were 10 cases of cervical cancer, 8 cases of endometrial cancer, 2 cases of deep infiltrating endometriosis (DIE), 3 cases of cervical uterine fibroids, and 2 cases of carcinoma of cervical stump. All patients underwent laparoscopic surgery, including radical surgery for cervical cancer with sentinel lymph node biopsy (SLNB), staging surgery for endometrial cancer with SLNB, lesion resection surgery for DIE, myomectomy for uterine fibroids, and radical trachelectomy. The basic clinical and surgical characteristics of the patients are listed in Table 1.

**Table 1:** Clinical and surgical data of patients.

	Number of patients
Patient (*n*)	25
Age, y (median)	51 (35–68)
BMI (median)	23.67 (18.73–28.51)
Pelvic surgery history	
Yes	9 (36%)
No	16 (64%)
Degree of pelvic adhesion^a^	
Grade 0-I	15 (60%)
Grade II	7 (28%)
Grade III-IV	3 (12%)
Diseases	
Cervical cancer	10 (40%)
Endometrial cancer	8 (32%)
DIE	2 (8%)
Cervical uterine fibroids	3 (12%)
Cervical stump cancer	2 (8%)
Intraoperative blood loss, ml (median)	100 (30–700)
Surgery duration, min (median)	235 (119–414)
Hospital stay, d (median)	14 (6–29)

^a^ Degree of pelvic adhesion: Grade 0: No adhesions. Grade I: Mild adhesions, with a small area of adhesion, surgical procedures easy. Grade II: Moderate adhesions, involving a wider area, surgical procedures complex. Grade III: Severe adhesions, with extensive adhesion areas, surgical procedures very complex. Grade IV: Extremely severe adhesions, almost impossible to distinguish structures, surgical procedures extremely difficult.

### 3.1 Fluorescence imaging and assessment

**Figure 1:**
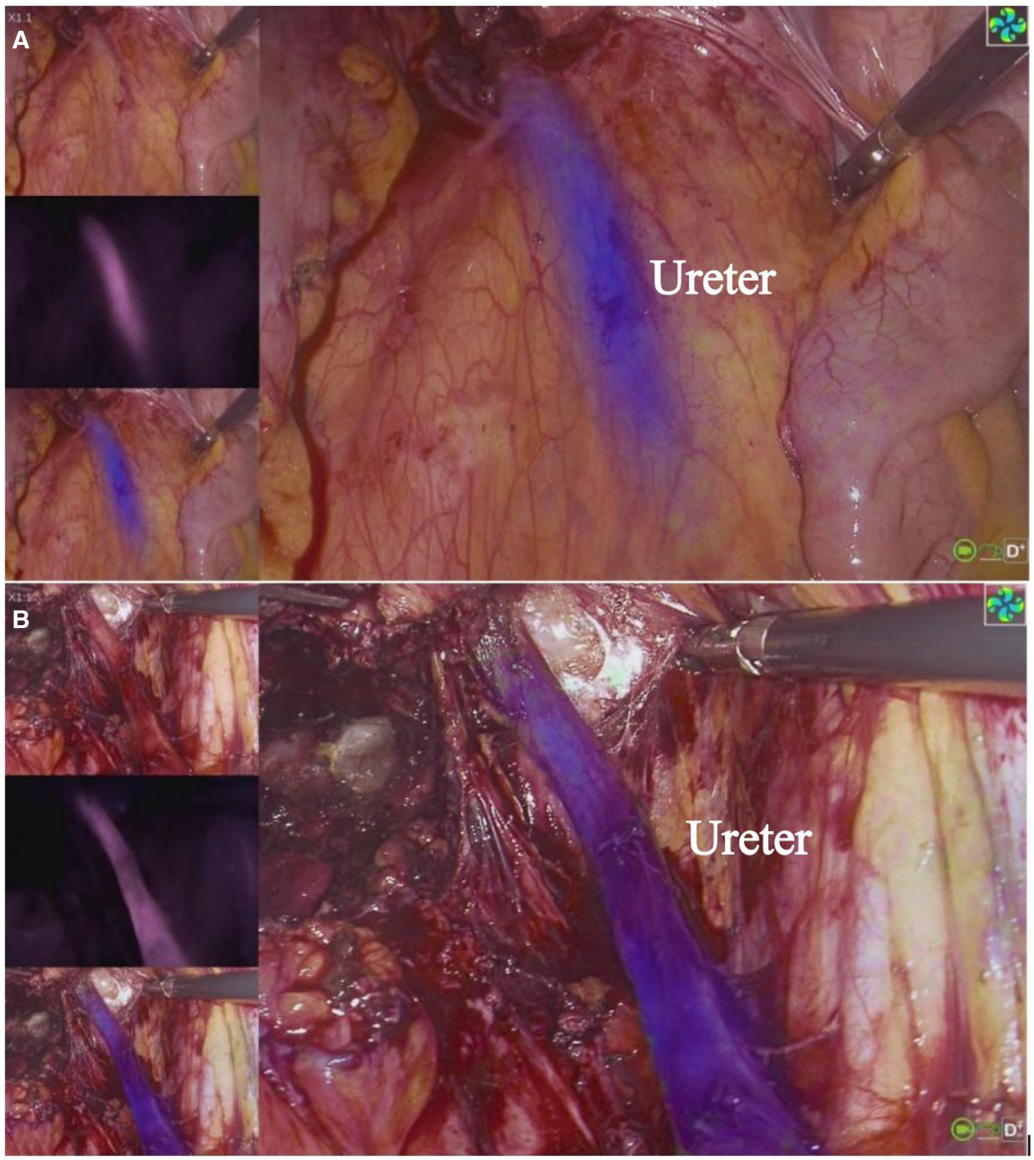
Visualization of ureter with MB in DIE. The three images on the left, from top to bottom, are white light image, fluorescent only black and white image and overlay of fluorescence (blue) to the white light image. The main screen displays the overlay image. In (**A**), the ureter is covered by the peritoneum and cannot be clearly observed under white light, and (**B**) shows the ureter can be clearly observed under both white light and fluorescence modes after it has been freed, and the position is consistent with the location shown in (**A**).

**Figure 2:**
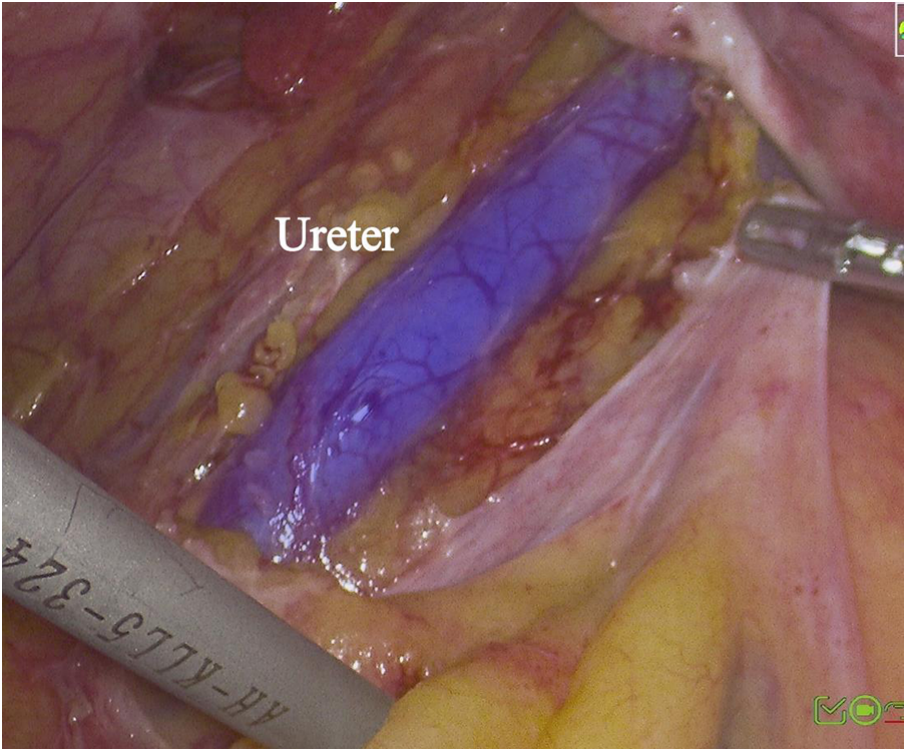
Visualization of ureter during laparoscopic removal of cervical leiomyoma.

**Figure 3:**
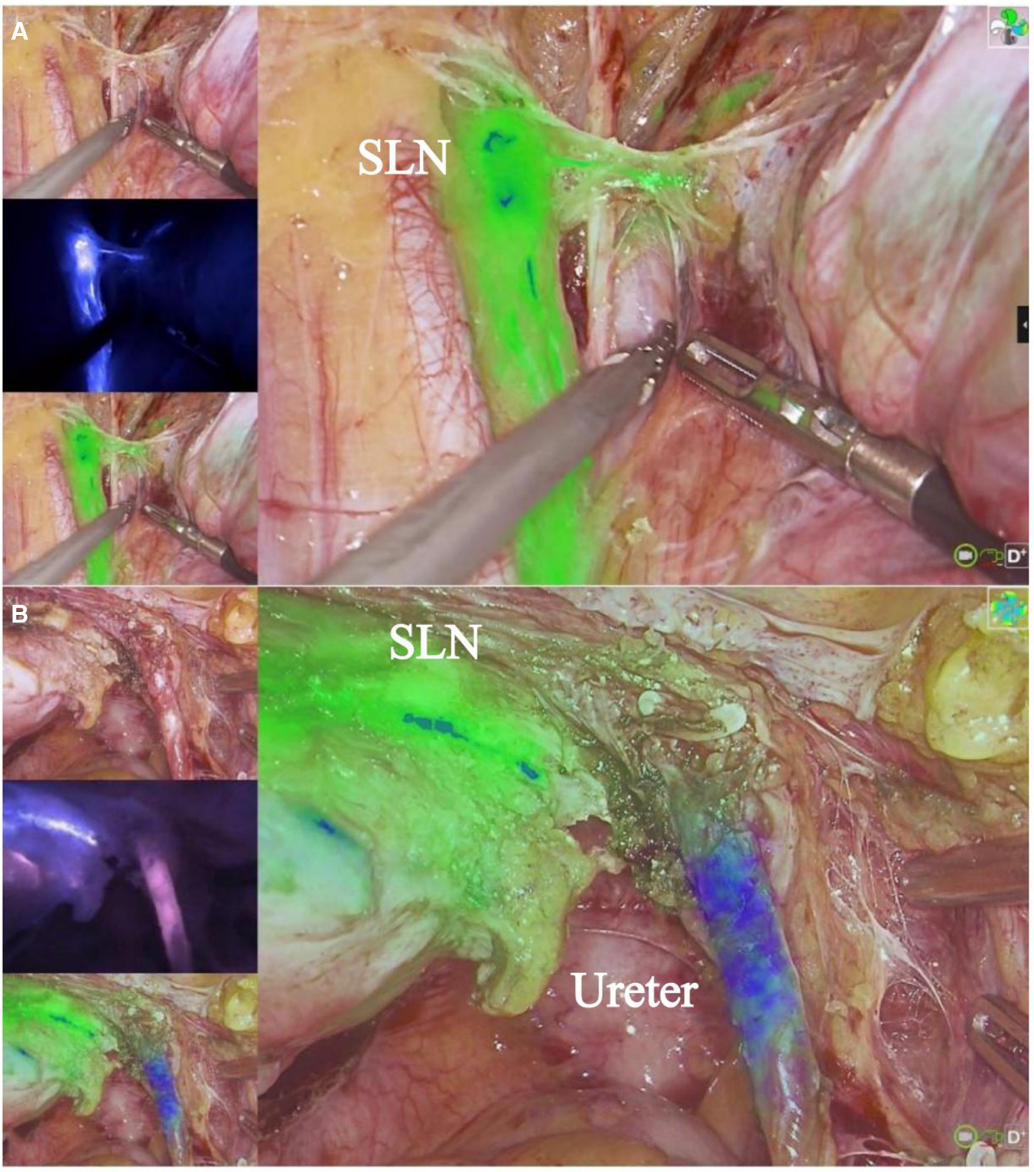
Radical surgery for cervical cancer and SLN. (**A**) shows ICG imaging of SLN. (**B**) shows MB imaging of ureter. The imaging system can simultaneously display both ICG and MB fluorescence.

Within 15–20 min after the initiation of intravenous MB drip, a total of 45 fluorescent ureters were observed in 25 patients, 2 cases did not exhibit obvious fluorescence in both ureters throughout the surgery, and in 1 case, the right ureter became visible after the administration of furosemide, while the left side did not show clear fluorescence. The remaining cases showed fluorescence in both ureters, and the visibility rate was 90%. We found that even though the ureters were covered by peritoneum, the ureters’ fluorescence was still visible. Upon opening the peritoneum, the location of the fluorescent ureters corresponded to the actual anatomy. Prior to opening the peritoneum and freeing the ureters, approximately 15 ureters were visible under white light (30%), whilst the rest were indistinct or invisible. In cases of DIE and cervical myoma, the ureters were clearly imaged, as shown in Figures 1, 2. And in cervical and endometrial cancer cases, both the SLN with green fluorescence and the ureters with violet-blue fluorescence were visible simultaneously, as shown in Figure 3. Among cases which both ureters exhibited fluorescence, 2 cases had a more pronounced fluorescence in the left ureter, 1 case had a more pronounced fluorescence in the right ureter. These three cases had varying degrees of ureteral incomplete obstruction and hydronephrosis due to lesion compression, and the affected side showing more pronounced fluorescence. It was observed that in cases with severe ureteral incomplete obstruction and hydronephrosis, the fluorescence persisted, while in cases without ureteral hydronephrosis, the fluorescence followed the flow of urine. And when the ureteral obstruction was relieved, fluorescence flows with the urine (Supplementary Videos 1, 2, Supplementary Video 1 shows fluorescence imaging in the surgery of cervical leiomyoma with ureteral incomplete obstruction and hydronephrosis, Supplementary Video 2 shows fluorescence imaging in the surgery of cervical cancer with normal ureters). The fluorescence image of the ureters could be observed as early as 5 min after the intravenous administration of MB, and the longest duration lasted for 4 h and 10 min. In this study, ureteral fluorescence was still observable in 16 cases (64%) at the conclusion of the surgery.

### 3.2 Adverse events

During surgeries, we observed 2 cases (8%) of decreased oxygen saturation, with the lowest level reaching 95%. After increasing the oxygen supply, the saturation quickly recovered. Throughout the surgical procedures, no adverse events related to MB occurred, such as hypotension, increased heart rate, arrhythmias, hemolysis, or allergies. No iatrogenic ureteral injuries, vascular or nerve damage, or other surgery-related complications occurred during surgeries (0%). Postoperatively, all patients had blue-green urine, which gradually subsided. There were no cases of renal function impairment or mortality after surgeries. We closely monitored potential postoperative adverse reactions which were possibly related to MB, including abdominal pain, distension, allergies (itching, etc), dizziness, headaches, nausea, vomiting, cardiovascular symptoms (chest tightness, palpitations, hypotension, arrhythmias), etc. 6 patients(24%) experienced the adverse reactions of grade 1 and 2, according to the Clavien-Dindo Classification (11). From February 2022 to December 2022, the same surgeon performed 78 laparoscopic procedures without the use of MB as patients had declined the intraoperative use of MB (Group B). The indications for surgery were radical cervical cancer surgery, endometrial cancer staging surgery, DIE lesion removal surgery, myomectomy, and hysterectomy. Ureteral injury occurred in 1 case (1.3%) due to dense adhesions at the distal end of the ureter, and a double-J stent was placed during the surgery. Postoperatively, 21 cases (26.9%) experienced the aforementioned adverse reactions of grade 1 and 2. Details of adverse events are presented in Table 2.

**Table 2:** Adverse events.

Adverse events	Group A (*n* = 25)	Group B (*n* = 78)
Abdominal pain	3 (12%)	8 (10.3%)
Abdominal distension	5 (20%)	18 (23.1%)
Allergies	1 (4%)	0 (0%)
Dizziness	3 (12%)	10 (12.8%)
Headaches	1 (4%)	3 (3.8%)
Nausea and vomiting	4 (16%)	9 (11.5%)
Cardiovascular symptoms	0 (0%)	0 (0%)

Group A represents cases in which MB was used to visualize ureters during surgeries, while Group B represents cases from the same period and the same surgeon in which MB was not used during surgeries.

## 4 Discussion

In gynecological surgeries, iatrogenic ureteral injury rates can be as high as 1.5% (12). Traditionally, common ureteral stents (13), luminescent ureteral stents (5), ureteral fluorescence stents (14–16), or intravenous injection of radioactive substances (7) were used for ureteral localization. However, these methods have disadvantages such as infection or ionizing radiation. MB is cheap, and by using near-infrared fluorescence imaging technology, patient's ureter appears purple-blue. This technique enables real-time, *in vivo* visualization of the ureter's location without ionizing radiation, making it non-invasive. Ureters covered by the peritoneum could not be immediately identified under white light but were clearly visualized in fluorescence mode. In our study, 90% of the ureters were clearly visualized in fluorescence mode, which is significantly higher than in white light mode. So, in fluorescence mode, the ureters were more easily detected, which shortened the surgical time for ureteric localization and helped avoid ureteral injury. Direct visualization of the ureter facilitates ureterolysis and helps to minimise direct trauma, delayed thermal injury and damaging the inferior hypogastric nerve plexus which tends to run below the ureter. Additionally, it can assist surgeons in quickly identifying abnormal, variant, displaced, or ureters affected by adhesions and fibrosis due to multiple surgeries or pelvic radiotherapy. In this study, during the resection of pelvic lesions in patients with DIE, MB-enhanced ureteral visualization and clearly delineated its boundaries, aiding lesion removal. Patients with large cervical fibroids, which compressed one side of the ureter causing local displacement, underwent ureteral fluorescence imaging with intravenous MB, revealing a distinct purple-blue colour that facilitated observation of the ureter's course and prevented ureteral injury.

Currently, there is no standard consensus on the dosage for MB, and there is a slight dose discrepancy on the optimal MB dose according to various studies. Verdeek (17)reported that in open surgeries for cervical cancer and ovarian cancer, an dosage of 0.25 mg/kg was recommended. In studies related to laparoscopic or open colorectal surgeries, Polom (12) suggested a dosage of 0.5 mg/kg and Barnes TG (18) recommended 0.75 mg/kg, whilst Yeung (19) recommended 1 mg/kg. Our study found that in gynecological surgeries, the intravenous rapid infusion of 1.5 mg/kg of MB resulted in clear visualization of ureters without associated safety issues. However, further research is needed to determine the ideal dosage. Almost all ureters could be observed in 15–20 min after administration of MB. Therefore, our experience suggests that the optimal observation time is 15–20 min after intravenous MB infusion. We also observed that at this dosage, the fluorescence imaging duration of MB was relatively long, with over half of the cases still showing ureteral imaging at the end of surgeries. This prolonged visualization allowed surgeons to confirm whether ureteral injuries had occurred.

MB is metabolized by the kidneys and excreted in the urine, limiting its use in individuals with severe renal impairment. Additionally, ureteral peristalsis causes pulsatile urine flow, affecting the fluorescence signal which varies over time (20). This study revealed that in cases of incomplete ureteral obstruction, the severity of hydronephrosis is positively correlated with the intensity of MB fluorescence imaging, whilst slow peristalsis or reduced urine volume led to unclear imaging. We also found that the clarity of imaging could be improved by injecting one or two boluses of Furosemide (20 mg) during surgeries. However, a study by Matsui (21) reported that when diuretics were used in combination with MB, increased urine excretion did not affect the fluorescence of MB dye. Currently, there is also no standard for the use of diuretics, dosage, or related issues. Further research is clearly needed to clarify these matters.

In our study, some patients did not have ureteral peristalsis or urine volume problems, but resulting in weak or unclear imaging. This phenomenon might be associated with fluorescence quenching or renal metabolism. When using the fluorescence laparoscope's gain function, the majority of ureters became more clearly visualized. Therefore, when encountering such situations during surgery, using the gain function may be effective. A small number of ureters were not visualized during the surgery. Review of these cases found that the reasons for this included obesity, increased visceral fat, thickened tissues, and poor ureteral activity with low urine volume. For these patients, our subsequent research may explore whether increasing the dosage of MB or using diuretics can improve visualization.

Our study found that MB is effective and safe for visualizing ureters in gynecological laparoscopic surgery. There were no iatrogenic ureteral injuries during surgeries involving MB, and in contrast, similar surgeries performed by the same surgeon during the same period without using MB had a 1.3% rate of ureteral injury. The reported incidence of iatrogenic ureteral injuries in gynecological surgeries is 0.15–1.5% (22). We preliminary propose that ureteral MB imaging can reduce the occurrence of iatrogenic ureteral injury in gynecological surgeries. The incidence of postoperative complications was similar between these two groups. We consider that the “MB-related adverse reactions” observed postoperatively might be attributed to surgical and anesthetic factors rather than an apparent association with MB. We acknowledge that our study has limitations, due to the small sample size and its short duration, therefore further clinical research is needed to validate its efficacy and safety.

We summarized previous studies on intraoperative ureteral visualization with either ICG or MB (Table 3). The studies indicated that using ICG for ureter visualization had a success rate of 100%, but it required the assistance of ureteral catheters. And in colorectal surgeries, with intravenous MB, the overall ureteral visibility rate ranged from 50% to 92.8%, with good safety. However no definitive clinical recommendations or standards have been established yet. Our study is the only one to report the use of MB for fluorescent ureteral imaging in gynecological laparoscopic surgeries, and has achieved satisfactory results.

**Table 3:** Previous studies of intraoperative ureteral visualization.

Reference	Dye type	Surgical specialism	Surgical procedure	Administration	No. of patients	Success rate	Associated complications
Siddighi (23)	ICG	Gynecologic	Laparoscopic	Ureteral catheter	> 10	100%	None
Lee (24)	ICG	Urologic	Laparoscopic	Ureteral Catheter and/or PNT^a^	25	100%	Yes, in one patient
Verbeek (17)	MB	Abdominal	Open	Intravenous	12	100%	None
Al-Taher (25)	MB	Colorectal	Laparoscopic	Intravenous	10	50%	None
Yeung (19)	MB	Colorectal	Both	Intravenous	8	90.9%	None
Barnes (18)	MB	Colorectal	Both	Intravenous	40	92.8%	None
Polom (12)	MB	Colorectal	Both	Intravenous	12	91.6%	None

^a^ PNT, percutaneous nephrostomy tube.

We believe this technique can be widely applied in future gynecological laparoscopic surgeries. Moreover, our study explored, for the first time, the dual-dye technique of ureteral MB fluorescence imaging (for the ureters) along with ICG fluorescence imaging for sentinel lymph nodes (SLN) in cervical or endometrial cancer, using the same camera system simultaneously, and this shows promising results. This dual imaging technique holds significant promise for patients requiring SLN biopsy and mapping, allowing surgeons to precisely remove lymph nodes while simultaneously avoiding ureteral damage. We are currently conducting a prospective clinical trial, and we expect to obtain more comprehensive results and clinical recommendations in the future.

## 5 Conclusions

Our initial study presents the application of ureteric MB fluorescence in gynecological surgeries. We visualized two fluorophores simultaneously through one camera system for the first time and achieved favorable outcomes. Our research demonstrates that intravenous infusion of MB can facilitate laparoscopic visualization of the ureters in gynecological surgeries under non-invasive and radiation-free conditions. This technique reduces surgical complexity, particularly benefiting less experienced surgeons by significantly minimizing the risk of ureteral injury and decreasing overall surgical duration. Furthermore, our initial experience is that this technique does not increase the risk of intraoperative or postoperative adverse events, indicating the safety of this technology. However, there is currently no clear consensus on the optimal dosage of MB or the use of diuretics. These questions necessitate further clinical investigation.

## Data Availability

The raw data supporting the conclusions of this article will be made available by the authors, without undue reservation.

## Supplementary material

Supplementary Video 1 MB fluorescence imaging of ureter in the surgery of cervical leiomyoma with ureteral incomplete obstruction and hydronephrosis.

Supplementary Video 2 MB fluorescence imaging of ureter in the surgery of cervical cancer with normal ureters.
